# COVID-19 Adaptive Interventions: Implications for Wellbeing and Quality-of-Life

**DOI:** 10.3389/fpsyg.2022.810951

**Published:** 2022-03-17

**Authors:** Haywantee Ramkissoon

**Affiliations:** ^1^College of Business, Law & Social Sciences, Derby Business School, University of Derby, Derby, United Kingdom; ^2^School of Business & Economics, Faculty of Biosciences, Fisheries & Economics, The University of Tromsø – The Arctic University of Norway, Tromsø, Norway; ^3^College of Business & Economics, Johannesburg Business School, University of Johannesburg, Johannesburg, South Africa; ^4^Faculty of Social Sciences and Leisure Management, Taylors University, Subang Jaya, Malaysia; ^5^Excelsia Business School, Sydney, NSW, Australia

**Keywords:** COVID-19 pandemic, adaptive interventions, nature social bonding, digital social bonding, place attachment, psycho-social, wellbeing, quality-of-life

## Abstract

Social bonds may assist in cultivating a more positive attitude to life through commonly shared meanings about the COVID-19 pandemic. The key challenge, however, is how to foster social bonds meeting the changing demands in a post pandemic world. Yet, it is in the middle of a crisis that the conversation needs to start about how to strategically plan for the recovery. This is important not only in the current pandemic, but also in a post pandemic world. Reinforcing or fostering new social bonds is likely to bring positive experiences. The latter is central to human health and wellbeing, and has potential to contribute greatly in enhancing people’s quality of life. In an attempt to foster place social bonding in the COVID-19 pandemic and beyond to subsequently contribute to wellbeing, this paper develops and proposes a new conceptual framework suggesting the need for adaptive social bonding interventions in the SARS-CoV-2 pandemic. This is an essential measure to manage the significant impacts on our global health services due to a decline in people’s mental health in addition to COVID-19 physical impacts. The paper discusses how promoting adaptive social bonding interventions (psycho-socio, digital and nature social bonding) can make people more resilient. It further discusses how they can be empowered psychologically, socially, and emotionally in the current challenging times. The conceptual framework posits that social bonding interventions can assist in maintaining better mental, physical, emotional, and spiritual wellbeing and discusses how these wellbeing outcomes may also be experienced post the pandemic. This has important benefits and is of relevance to governments, policy makers and healthcare professionals in delivering better health care and equipping people with coping mechanisms both throughout the pandemic and in the long run.

## Introduction

The SARS-CoV-2 virus continues to impact negatively on people’s quality of life (Ramkissoon, 2020a,b; Zhang and Ma, 2020; Desai et al., 2021; Raman et al., 2021). COVID-19 physical and mental health impacts had health leaders, policy makers, front-liners, and other national and global co-actors constantly working hard to attend to the unprecedented challenges brought by the rapid spread of the virus (Holmes et al., 2020; Ramkissoon, 2021a; Su et al., 2021). This mini review summarizes multidisciplinary evidence from literature showing the need for adaptive social bonding interventions during the COVID-19 pandemic. This has important implications both in the current and post the COVID-19 pandemic to promote people’s wellbeing and quality of life.

The SARS-CoV-2 pandemic brought negative emotions such as anger and sadness caused by sickness, loss of close ones, and bereavement (Aslam et al., 2020; Pfefferbaum and North, 2020). COVID-19 also posed serious concerns to infected patients with pre-existing cardiovascular diseases (Li et al., 2020; Mai et al., 2020; Tadic et al., 2020). In addition, the economic impacts brought a rise in people being unable to cope with their existing medical conditions fueling other issues including domestic violence, drugs, and alcohol among others. Suicidal tendencies have been on the rise (Gunnell et al., 2020; McIntyre and Lee, 2020; Pfefferbaum and North, 2020, Sher, 2020). Loneliness has been made worse by the COVID-19 pandemic and remains a chief health concern and demands that agencies develop interventions to build resilience for various groups in society (Ramkissoon, 2021a,b). We need innovative and reinforcing systems to be put in place to better cope with the above-mentioned challenges post the pandemic. We need to consider important mechanisms to promote the wellbeing and enhance quality of life for all of our people to prepare for the future. This will require global collaborative efforts from stakeholders.

There continues to be increasing demand for healthcare organizations to address the detrimental effects of COVID-19 on mental health and wellbeing; healthcare staff have been reporting anxiety symptoms (Siddiqui et al., 2021; Yıldırım et al., 2021). In this ongoing crisis situation, psycho-education interventions, relaxation and meditation techniques, and spending time in nature are recommended to healthcare staff and other COVID-19 frontliners. These techniques can promote social bonding and bring important restorative effects on human health and wellbeing.

Another group of the society which has been significantly impacted are elderly populations (Patel and Clark-Ginsberg, 2020). Many governments strongly discouraged elderly mobility during the COVID-19 which could have contributed to higher levels of loneliness and depression impacting on their quality of life (Liu et al., 2021). The COVID-19 traumatic event continues to add pressure on the healthcare systems demanding that we find additional and complementary means of support for those in need.

Social bonding has been defined as the social closeness required to experience a sense of belongingness in the community (Wolf et al., 2016). Social bonds are characterized by meanings that people commonly assign in settings emphasizing the human–place bond. The latter is commonly defined as place social bonding depicting the bond people share in a common space (Ramkissoon et al., 2013a,b). Social bonding can play a significant role in helping others regulate their emotions in both the pandemic and post-pandemic times. While it’s in human nature to bond with one another, people do tend to rely further on their social relations in a time of crisis (Ahmed et al., 2020; Jetten et al., 2020). During the COVID-19 pandemic, people have had to confine to their homes and become overly dependent on their social supports within their home environments. The social isolation from their social groups and an over dependence on the people they live could lead to household conflicts (Behar-Zusman et al., 2020) and impact mental health and quality of life (Xie et al., 2020; Alzueta et al., 2021). Promoting social bonding can help to promote better coping skills to deal with the pandemic (Ahmed et al., 2020; Ramkissoon, 2021b).

Social bonds may assist in cultivating a more positive attitude to life through commonly shared meanings about the pandemic. The key challenge however is how to foster social bonds meeting the changing demands in a post pandemic world. Yet, it is in the middle of a crisis that the conversation needs to start about how to strategically plan for the recovery. This is important not only in the current pandemic, but also in a post pandemic world. According to the Broaden-and-Build theory, people experiencing overall mental wellbeing will be more resilient and also more flexibly adapt their emotional states to changing life circumstances (Cohn et al., 2009; Prinzing et al., 2020). Reinforcing or fostering new social bonds is likely to bring positive experiences. The latter is central to human health and wellbeing, and has potential to contribute greatly in enhancing people’s quality of life (Farmer and Kashdan, 2012; Ramkissoon et al., 2018; Ramkissoon, 2016, 2020a). Promoting social bonding can also make people more resilient, they could be empowered psychologically, socially, and emotionally in the current challenging times and maintain better mental, physical, emotional and spiritual wellbeing (Ramkissoon, 2021a). These wellbeing outcomes may also be experienced post the pandemic.

An important discussion to bring on the table is the need to promote social bonding interventions for people to cope with the pandemic and adjust to the post-pandemic world. Promoting pro-social attitudes and behaviors can foster wellbeing and quality-of-life outcomes (Ramkissoon et al., 2018; Townsend et al., 2018; Ramkissoon, 2022). This has important benefits and is of relevance to governments, policy makers, and healthcare professionals in delivering better health care and equipping people with coping mechanisms both throughout the pandemic and in the long run. In an attempt to foster place social bonding in the COVID-19 pandemic and beyond and subsequently contribute to wellbeing, this paper develops and proposes a new conceptual framework suggesting the need for social bonding interventions in the SARS-CoV-2 pandemic. This is an essential measure to manage the significant impacts on our global health services due to a decline in people’s mental health in addition to COVID-19 physical impacts (Holmes et al., 2020; Pfefferbaum and North, 2020; Ramkissoon, 2021c).

## Literature Review

Wellbeing has been defined as “each time an individual meets a challenge, the system of challenges and resources comes into a state of imbalance, as the individual is forced to adapt his or her resources to meet this particular challenge” (Kloep et al., 2009, p. 337). While wellbeing has been well studied in literature, the concept continues to attract significant interest from scholars, psychological practitioners and complementary, traditional and integrative medicine providers in the current pandemic. The broader societal goal is to collectively contribute and address the recurring calls for actions to maintain global public health and wellbeing. Scholars argue that the interaction between the brain, mind, body, and human behavior contribute in reducing stress, promoting a general sense of wellbeing and enhancing quality of life (Ramkissoon, 2022). Scholars have been exploring interventions in alternative and integrative medicine to foster and maintain social bonds (Behan, 2020; Ramkissoon, 2021a). These include psycho-socio therapy, psycho-education, relaxation, and nature therapy; the goal is to promote place social bonding for people’s mental, physical, emotional, and spiritual wellbeing. The latter can promote a general sense of wellbeing and in turn enhance quality of life outcomes. Studies have shown that improving residents’ quality-of-life remains an important outcome for several nations (Uysal et al., 2020; Berbekova et al., 2021; Wang et al., 2022). Place confinement as result on the limitations of daily life activities have had serious implications on people’s quality-of-life. The prohibition of meeting with others have contributed in making people more vulnerable and hence the need for more social support (Ferreira et al., 2021). Defining quality-of-life continues to pose a challenge for researchers. The concept has numerous definitions in literature which are context-dependent (Post, 2014). The Center for Disease Control and Prevention (2022) defined quality-of life as a person or group’s perceived physical and mental health over time.

**FIGURE 1:**
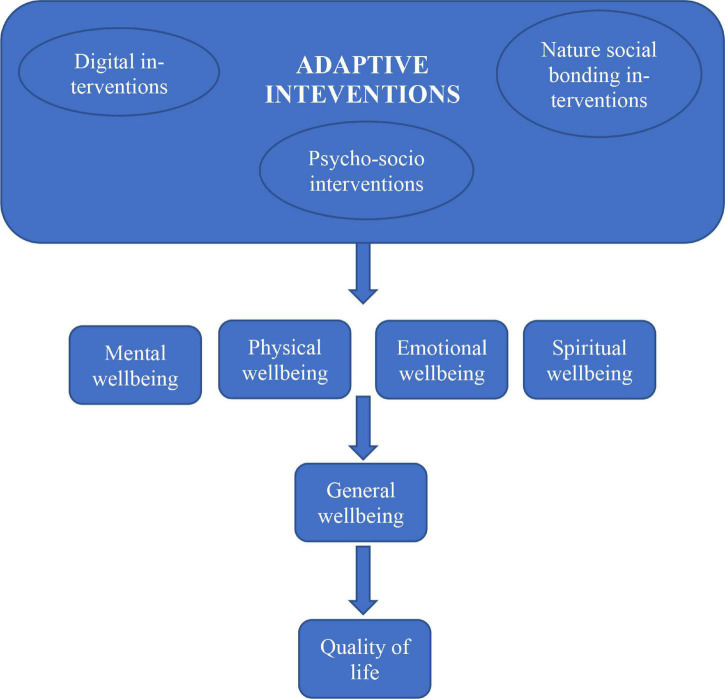
The proposed theoretical framework.

Using the COVID-19 place confinement as the context, a single integrative model of adaptive social bonding interventions (psycho-social, nature, and digital), wellbeing and quality of life is developed and proposed (Figure 1). The premise is about individuals being equipped with the physical, social, and psychological resources when faced with the challenges such as the COVID-19 pandemic. The proposed conceptual framework draws on multidisciplinary research streams including traditional health, and complementary and integrative medicine, behavioral and environmental psychology, and sociology (e.g., Bavel et al., 2020; Tillu et al., 2020; Ting et al., 2020; Ramkissoon, 2021a,b, 2022). The associations between the constructs under investigation are depicted by arrows in the conceptual framework.

## Psycho-Socio Interventions

Psycho-education techniques have been used to deal with psychological impacts following earthquakes (Oflaz et al., 2008). During the COVID-19 pandemic, there has also been a range of programs developed and recommended in complementary and integrative medicine to assist treatment of COVID-19 patients’ recovery and those impacted by place confinement (Cheng et al., 2020; Ramkissoon, 2020a; Zhou et al., 2020). Emphasis needs to be drawn on the benefits of social bonding intervention programs for everyone in a community. For instance, social bonding interventions programs targeted at dealing with post-traumatic events need to be developed across multiple groups e.g., children, adolescents, adults, and the elderly. This is an important recommendation for policy makers and health leaders as we need to be better equipped with more evidence-based findings to better deal with the post COVID-19 climate. Social bonding interventions in COVID-19 can build on Roberts (2005) crisis intervention model where a seven-step strategy is adopted to help people cope with a crisis. The seven steps include: (1) explore resilience and coping abilities based on risk factors; (2) rapidly establishing rapport with the person; (3) assess risks based on evidence as opposed to the person’s perceived risk based on values and beliefs; (4) dealing with emotions appropriately; (5) identify alternatives for shared decision making; (6) implement action plans that the person can understand and manage; and (7) following up to allow further interaction and cultivate a sense of ‘being there’ for the person (Sethuraman, 2020).

In the COVID-19 and post the pandemic, many patients could benefit from psychosocial therapy. These psycho-social interventions are designed to promote health and wellbeing outcomes (Umberson and Karas Montez, 2010; Gühne et al., 2015). Examples of psycho-social therapy interventions can include cognitive behavioral therapy psycho-educational therapy, relaxation therapy, and meditation therapy (Ramkissoon, 2021b). There could be an increase in more suicidal tendencies which will require care from mental health professionals and possibly psychiatric treatment at the hospital (Pfefferbaum and North, 2020). Medical doctors and behavioral psychologists argue that suicidal tendencies can result from the death of loved ones, bereavement, unemployment, and economic crisis (Pfefferbaum and North, 2020; Ramkissoon, 2021a,b). Psycho-social interventions include therapeutic landscapes to address patients’ COVID-19 emotional distress (Duan and Zhu, 2020; Majeed and Ramkissoon, 2020; Ramkissoon, 2021a). There has also been a decline inability to cope with existing medical conditions e.g., cancer, drugs, alcohol, domestic violence among other issues while being place confined during the COVID-19 pandemic. The media reporting the COVID-19 pandemic news has also can be emotionally draining demanding that patients’ exposure to such sources of information be closely monitored (Pfefferbaum and North, 2020). Complementary, traditional, and integrative medicine providers can assist with the above by offering stress management and coping strategies. Examples of emotional distress can be from the news channels. Children feeling isolated from their school friends and peer groups (Peguero et al., 2016; Xie et al., 2020) also need to be supported through psycho-social therapy. This can possibly be effected online by behavioral psychologists and healthcare professionals. Clowning can be used to cheer children in hospitals (Dionigi et al., 2018) to reduce their negative increase their positive emotions. Ruffolo et al. (2005) in their study on children with severe emotional disturbance recommended multiple family group psycho education to foster support, education and empowerment. The family group psycho interventions recommended by Ruffolo et al. (2005) focused on collaborative efforts for relationship building, educational tools to control and manage symptoms, the coping skills and information sharing. Their intervention model was a parent/professional model promoting parental support, knowledge of their child’s illness and symptoms, empowerment, and their advocacy skills (see Ruffolo et al., 2005).

Financial, social, and health and wellbeing impacts of the SARS-CoV-2 global pandemic is likely to last beyond the pandemic. Interventions are needed to re-build people’s confidence. Scholars argue that trust among strangers and working through cross-cultural differences may be key to managing the COVID-19 pandemic (Romano et al., 2021). Trust has been evidenced to be an important factor in building capacity and resilience in the community during crises (Crouse Quinn, 2008; Limaye et al., 2020; Opel et al., 2020; Harring et al., 2021). Trust remains an important tenet of authentic community investment (Ojikutu et al., 2021); it is imperative to focus on fostering trust among social groups (Ramkissoon, 2020a) and invest build their psychological, social, technological, and economic empowerment. Perceived empowerment originates from the community psychology literature defined by Rappaport (1987) as securing authority over one’s life through engagement in collective activities through democratic participation. Zimmerman (1995) has since defined empowerment as “efforts to gain control, access to resources, and a critical understanding of one’s sociopolitical context” (Zimmerman, 1995, p. 583). Multiple dimensions of empowerment including psychological, social, political, and economic dimensions and few others have been studied in the community psychology literature and across disciplines (e.g., Laverack, 2001; Nunkoo and Ramkissoon, 2016). A recent study by Aleshinloye et al. (2021) evidence that psychological empowerment is the most significant dimension of resident empowerment influencing place attachment suggesting that people have associated meanings with their place for which they hold great value. Studies have further evidenced that people who are empowered through jobs bringing economic benefits are likely to experience higher wellbeing outcomes which in turn positively influence their quality of life (Woo et al., 2018). Social empowerment is also one of the most prominent dimensions of empowerment; it is defined by collective behaviors (Ramkissoon et al., 2018), strengthening a community’s sense of cohesion and integrity (Scheyvens, 1999). Social empowerment plays a significant role in generating wellbeing benefits; this demands a deeper focus on social bonding and wellbeing post pandemics and crises (Ramkissoon, 2020b). Scholars argue that the more empowered people are in a community, the greater will be their standard of living and their overall life satisfaction, happiness and fulfilment (Andereck and Nyaupane, 2011; Aleshinloye et al., 2021). Place attachment, defined as the emotional bonding between a person and place has been associated with improved quality of life (Ramkissoon et al., 2018). The concept has been widely studied in environmental psychology with researchers now agreeing that it is multi-dimensional in nature (Scannell and Gifford, 2010). Ramkissoon et al. (2012) conceptualized place attachment as an overarching construct with sub-dimensions of place identity, place dependence, place affect and place social bonding. Place dependence and place identity have been the most prominent sub-constructs studied in place attachment research. In the COVID-19 pandemic context, it may be logical to expect that people would require more social support. The sub-construct of place social bonding plays a significant role in fostering pro-sociality when dealing with loss, separation and grief. An investigation of the association between people’s social bonding, their wellbeing and quality of life in the post pandemic context will be important to further inform policy for broader societal health outcomes.

## Nature Social Bonding Interventions

Understanding how fostering of social bonds in nature-based settings can impact on our health and wellbeing and the planet’s health is critical in the current challenging times of the COVID-19 pandemic. It has important relevance to many healthcare professionals and across disciplines of research and teaching. Health practitioners and behavioral scientists are recommending fostering of social bonds to confront challenges in safeguarding the planet’s resources and people’s wellbeing. Fostering people social bonds in environmental settings can contribute to quality of life aligning with the United Nations Sustainable Development Goals (Ramkissoon et al., 2013a; Ramkissoon, 2020a).

Green theory (Pretty et al., 2017) posits that people who care for their general health, body and self tend to have more affinity for nature. Ramkissoon (2021a) argues that body-mind interventions in nature can contribute to mental wellbeing with improved quality-of life benefits. Scholars have been showing increasing interest on the benefits of connectedness to nature and associated health and wellbeing benefits. Recent research stress the importance of spending time with others in nature-based settings (Triantafyllidis and Kaplanidou, 2019; Triantafyllidis and Darvin, 2021) discussing the collective meanings that can be created for a common purpose such as collective engagement in pro-environmental behaviors (Ramkissoon et al., 2013a,b, 2018). Literature also discusses the wellbeing benefits of nature social-bonding and how it can lead to new habits formation (see Ramkissoon, 2020a) for improved wellbeing and quality of life. Pretty et al. (2017) study found that different types of nature exposure including urban parks, home gardens, large biodiversity areas, and small and large landscapes contribute to wellbeing benefits across age groups.

There is a need to engage all groups in society in nature exposure. For instance, nature-based social bonding interventions can bring a range of health and wellbeing benefits to children. Designing interventions to integrate both children and teachers in nature-based programs can promote social bonding enabling them to collectively contribute and address societal, environmental and health issues. Children can develop affective ties with others in nature (Cheng and Monroe, 2012). This approach has potential to better equip our children to face the post pandemic new normal while fostering a deeper sense of responsibility as they grow up.

The pandemic has also left many with feelings of grief, separation, and loss. Often those who are depressed report a lack of social support (Matheson et al., 2005). In a post pandemic time, the elderly can meet with others in neighborhood parks/community green spaces and engage in low-effort pro-environmental (e.g., removal of weeds, light gardening activities) (Ramkissoon et al., 2013b) and pro-social behaviors (e.g., meeting with others in nature parks and provide emotional support) (Ramkissoon, 2020a,b). The emphasis needs to be on collective engagement in nature (Wolf et al., 2015; Wu et al., 2022), this has important implications for social inclusion leading to mental health (Tew et al., 2012), and physical and spiritual wellbeing benefits (Ramkissoon, 2021a). This finds support in Social Identity theory stressing on people’s sense of themselves as members of a social group (Alexander Haslam, 2014). People may get happier and have a higher sense of purpose when they are in groups (Iyer et al., 2009). Nature social bonding interventions need to focus on co-creation of collective meanings. It’s important to note that people coming together in shared environmental settings can be from diverse groups. Social norming interventions can be further facilitated through value creation and a collective purpose (Meeussen et al., 2018; Ramkissoon and Uysal, 2018); a common purpose can trigger collective interest. This in turn can generate more group commitment and trust (Nunkoo and Ramkissoon, 2012; Nunkoo et al., 2012; Ramkissoon, 2020b) leading to pro-sociality and wellbeing.

## Digital Interventions

In the current SARS-CoV-2 pandemic, one of the most important strategies being recommended is online social bonding or ‘digital bonding’ as we maintain physical distancing (Bavel et al., 2020; Ramkissoon, 2020a). The social confinement as a result of physical social distancing caused a heavy blow to people across many societies. People are being encouraged to meet more frequently using social media. This has also triggered new social connections over the internet to discuss issues of common interests. Digital communication with others can help face problems of social isolation. Past studies have shown that through pro-social behaviors e.g., assisting others to regulate their emotions, an individual can also help regulate one’s own emotions and decrease feelings of depression (Doré et al., 2017).

A significant drawback of digital engagement is that people can also aggravate their stress levels. Recent research has discussed negative impacts of media on people’s mental health (Keles et al., 2020). Health professionals are recommending against constant exposure to distressing news of COVID-19 (Xiong et al., 2020) claiming that the COVID-19 panic is traveling faster than the pandemic and causing psychological distress and impacting on adults’ mental health (Haider et al., 2020). Behavioral experts suggest that engagement in discussions with social media contacts with negative emotions might not help in promoting positive emotions and reinforce their pandemic coping skills (Ramkissoon, 2020a). It is imperative that the exposure to sources of distress be minimized among groups which are highly sensitive to the media news as this may produce more damaging impacts on their mental health. Social media interactions can also become addictive and trigger a dependence on technology for social bonding. Failure of technological platforms such as Zoom can trigger negative emotions and bring further psychological distress impacting on individuals’ wellbeing. In contrast however, those who are more resilient and willing to help and be helped may want to engage further with their peers on social media to support one another and promote a deeper sense of social connection and boost psychological wellbeing.

Th author recognizes that impacts of the digital divide have been heavily felt during the COVID-19 pandemic. Those without ready access to the internet have had missed opportunities as many essential activities moved online (Ramsetty and Adams, 2020; Lai and Widmar, 2021). For instance, telemedine has been the savior for many and yet fueled health inequality as it’s not available and accessible to all (Watts, 2020). Enforced closures have promoted teleworkability (work that can be done from home) leaving many at a disadvantage (Sostero et al., 2020). Sostero and colleagues study findings suggest that since the COVID-19 pandemic, the large expansion of telework is skewed toward high-paid white-collar jobs. Digital bonding is no exception as it is favored for those social classes who have access to technological tools.

Despite these challenges, fostering social bonds will remain important for individual and community health and wellbeing. Following the unprecedented COVID-19 health tragedy, social support will be required to promote mental health. Literature has evidenced that in a post disaster/crisis context, bereavement and grief may continue (Saltzman et al., 2020). This could be over the loss of close ones and other negative effects such as loss of employment and financial instability (Cattan et al., 2005; Valtorta et al., 2016; Nande et al., 2021). Loneliness and social isolation could aggravate for individuals suffering from depression impacting further on their general health and wellbeing. Some examples of negative spillovers of the pandemic have been conflicts, physical abuse, divorce as a result of place confinement (Ahmed et al., 2020). A lack of social support post traumatic events can lead to prolonged mental distress and trauma. Reinforcing social bonds within the family remains paramount. Social bonding interventions hence are needed to foster more social support through existing social bonds or/and creation of new social bonds. Figure 1 shows the proposed theoretical framework for social bonding interventions.

## Implications for Future Research and Conclusion

The COVID-19 pandemic has certainly been and continues to be a stressful time, However, it also provides a context to bring some important changes in the current societal systems. It provides a window of opportunity to foster meaningful social bonds that can be sustained over the long run. Fostering social bonding in such challenging times as in the pandemic can make governments, health professionals, businesses, other co-actors, and all residents think further and implement strategies that could deliver better societal outcomes through a genuine caring for people both in the home environment, educational institutions, place of work and in healthcare locally, nationally, and globally. The unprecedented challenges of COVID-19 could fuel more collective actions through social networks to achieve health and wellbeing and promote quality of life aligning with the Global Health Strategy.

The COVID-19 global health pandemic has and will bring radical changes in people’s ways of life post the pandemic. The importance of staying socially bonded through interventions designed to maintain and promote mental wellbeing and foster deeper social bonds in a post pandemic context needs to be reinforced. Social bonds are important in dealing with pandemics’ prolonged economic, social, health and wellbeing negative impacts. In preparing for the post-pandemic, we could also use the current COVID-19 as a context providing an opportunity to re-design and accommodate the needs of the aging population. Re-designing neighborhoods with an aesthetic appeal is likely to lead to better health outcomes for the elderly.

Evidence suggests that elderly people who find their social and environmental surroundings to be pleasant can get physically more active through engagement in activities (Zhai et al., 2021). Elderly people are more motivated to move out of their homes to enjoy the aesthetic environment outdoors which have important health and wellbeing outcomes (Feng et al., 2020). They are likely to develop deeper social bonds in their place surroundings which can enhance their overall health and wellbeing. The social place can also represent the values which need to be shared with the present and passed on to the future generations. Promoting place social bonding can generate important benefits for healthy aging in place both in the immediate and long term. These values also promote affective ties to a place and assist in personal development through positive affect. Promoting place social bonding is recommended to urban planners, policy makers and healthcare leaders and other co-actors to promote global health and wellbeing aligning with the UN Sustainable Development Goals.

The COVID-19 Pandemic also provides an opportunity to think further on the working class and preparing for the post pandemic world. The “work from home” culture has been well established in several companies as most people had to be restricted to their homes to control the SARS-CoV-2 virus (Bick et al., 2020). It is quite likely that many will get back to the pre-COVID 19 office environment or maintain both the “work from home” and “at the office” models post the pandemic (Spurk and Straub, 2020). It is imperative that we design interventions to further integrate healthier practices in the workplace. This can include pro-social and pro-environmental activities at work (Ramkissoon, 2021a).

Education plays a significant role in preparing for better societies. It has been largely apparent from the report from the World Organisation for Early Children Education (OMEP) that adults tend to underestimate competencies of children in fostering sustainability outcomes (Engdahl, 2015). Recommendations were made to foster education for sustainability which can be a key driver of quality childhood education. Educators need to continue working on fostering behaviors that will encourage healthier lifestyles starting from the kindergarten. Designing social bonding interventions to integrate both children and teachers in programs to enable them to collectively contribute and address societal, environmental and health issues will better equip our children to face the post pandemic new normal while fostering a deeper sense of responsibility as they grow up.

During the COVID-19 pandemic, researchers have been looking at how social bonding can engage people so they can cope with the pandemic stress and social isolation and contribute to wellbeing and generate quality of life outcomes. The argument is that with the necessary support, residents can learn to better cope with the stress and grief they experience with loss and adapt in the post pandemic world. However, there is always the danger that we forget the trauma. Social bonding interventions designed taking into account contextual needs will need to be closely monitored and evaluated.

Further, while interventions are being recommended in disaster management and recovery programs (Taylor et al., 2012) and as a COVID-19 pandemic response tool, more evidence-based findings are required to gauge its effectiveness in fostering meaningful social bonds (Ramkissoon, 2020a). We also need to develop newer methods and interventions that help people to connect and re-connect post the pandemic to combat loneliness, boredom and psychological distress.

## Author Contributions

HR is the sole author of this manuscript.

## Conflict of Interest

The author declares that the research was conducted in the absence of any commercial or financial relationships that could be construed as a potential conflict of interest.

## Publisher’s Note

All claims expressed in this article are solely those of the authors and do not necessarily represent those of their affiliated organizations, or those of the publisher, the editors and the reviewers. Any product that may be evaluated in this article, or claim that may be made by its manufacturer, is not guaranteed or endorsed by the publisher.
